# Using a gender lens to understand eating behaviours of adolescent females living in low‐income households in Bangladesh

**DOI:** 10.1111/mcn.12841

**Published:** 2019-06-20

**Authors:** Lauren S. Blum, Rasheda Khan, Marzia Sultana, Nahian Soltana, Yasmin Siddiqua, Rudaba Khondker, Sabiha Sultana, Alison Tumilowicz

**Affiliations:** ^1^ Global Alliance for Improved Nutrition (GAIN) House 20, Road 99, Level 4, Gulshan 2 Dhaka 1212 Bangladesh; ^2^ The Nielsen Company (Bangladesh) Ltd. Dhaka Bangladesh

**Keywords:** adolescent females, Bangladesh, food intake, gender discrimination, qualitative research, undernutrition

## Abstract

Adolescence is a critical period characterized by rapid physical, psychological, and social development and growth. In Bangladesh, high rates of undernutrition persist among adolescent females living in low‐income households. Prevalence of adolescent marriage and pregnancy is extremely high, with almost half of Bangladeshi women giving birth by 18 years of age. Qualitative research was carried out from April to June 2017 to examine individual, social, and environmental factors influencing eating behaviours of female adolescents between 15 and 19 years of age living in low‐income families in urban and rural settings in Bangladesh. Methods included freelisting exercises (33), key informant interviews (11), in‐depth interviews (24), direct observations (16), and focus group discussions (12). Findings show that household food insecurity necessitates adjustments in meal food quality and frequency. Gender norms prescribe that females receive small meal portions and make sacrifices in food consumption so that male family members can eat more. Work and school schedules cause long breaks between meal consumption, restricting food intake of adolescent females for extended periods. Gender discrimination and its manifestations likely amplify susceptibility to psychological stresses in adolescent females. An inferior social position makes adolescent females living in food insecure households vulnerable to undernutrition, with factors affecting food deprivation increasing as they approach childbearing. Policies to increase age of marriage and reduce adolescent pregnancy must continue. Programmes must ensure that school‐going adolescents eat adequately during the school day. Prolonging school education and strengthening the economic viability of women should alter cultural expectations regarding marriage age and normative female roles.

**Key messages**

- Gender norms interact with economic, social, and structural factors to make female adolescents vulnerable to poor dietary intake and undernutrition.
- Female socialization involves making sacrifices related to food consumption so that male family members can eat more.
- Work of family wage earners and school schedules foster long breaks between meals, causing adolescent females living in low‐income households to skip meals
- Gender discrimination and its manifestations appear to amplify psychological stresses in adolescent females living in food insecure households.
- Prolonging school education and strengthening the economic viability of women should alter cultural expectations regarding normative female roles in households and society.

## 1 INTRODUCTION

Adolescence is a transitional period from childhood to adulthood characterized by rapid physical, psychological, and social development and growth (Das et al., 2017). Early physiological changes involve puberty and sexual development and, in later phases, pubertal maturation (Das et al., 2017). Adolescents experience significant physical growth accounting for about 50% of adult weight and 15% of adult height (Spear, 2002). These dramatic physical changes create increased demands for nutrients and energy (Das et al., 2017). Simultaneously, adolescents undergo psychosocial changes, including growing concerns about physical appearance, greater autonomy, and modifications in roles and responsibilities; all of which can impact on food intake and nutritional status (Das et al., 2017; Story, Neumark‐Sztainer, & French, 2002). Research shows that adolescents frequently have poor eating habits, increasing risks for iron deficiency anaemia, micronutrient deficiencies, suboptimal linear growth, and undernutrition, as well as overweight in many contexts (Akseer, Al‐Gashm, Mehta, Mokdad, & Bhutta, 2017; Christian & Smith, 2018). Poor adolescent eating behaviours and undernutrition are predictive of adult eating practices and health (Larson, Neumark‐Sztainer, Hannan, & Story, 2007; Pedersen, Holstein, Flachs, & Rasmussen, 2013). Determinants of nutritional problems vary according to the sociocultural and economic context and physical environment where adolescents live.

Despite rising incomes and access to a variety of foods, widespread malnutrition persists in low‐ and middle‐income countries, particularly in urban slums and rural areas (Abarca‐Gómez et al., 2017; Pörtner & Su, 2018). In these settings, girls are often more likely than boys to drop out of school at a young age, increasing the likelihood of social isolation, early marriage, and childbearing when girls are still maturing (Gibbs, Wendt, Peters, & Hogue, 2012; WHO, 2014a). Adolescent mothers are at greater risk for severe delivery complications, with nutritional status prior to conception critical for maternal and infant outcomes (Black et al., 2013; Han, Mulla, Beyene, Liao, & McDonald, 2011). An early start to childbearing has been shown to trigger negative health and social consequences, perpetuating the intergenerational cycle of malnutrition (Branca, Piwoz, Schultink, & Sullivan, 2015; Temmerman, Khosla, Bhutta, & Bustreo, 2015; WHO, 2011, 2014b).

For decades, undernutrition has been a major public health problem in Bangladesh, with the prevalence of malnutrition historically reported to be higher among females than their male counterparts (Chen, Hug, & D'Souza, 1981; Choudhury, Hanifi, Rasheed, & Bhuiya, 2000). Recent data show that 31% of ever‐married females aged 15–19 are underweight (BMI < 18.5), and 26% of adolescent girls 10–18 years are stunted (Helen Keller International [HKI] & BRAC Institute of Global Health [BIGH], 2014; Helen Keller International [HKI] & James P. Grant School of Public Health [JPGSPH], 2014, 2016; National Institute of Population Research and Training [NIPORT] et al., 2016). Over half of adolescent girls and women are reported to consume inadequately diverse diets (Helen Keller International [HKI] & James P. Grant School of Public Health [JPGSPH], 2014), with more than 70% of kilocalories coming from rice and intake of animal source foods limited (Leroy, Ruel, Sununtnasuk, & Ahmed, 2018). Energy intake is also inadequate (Leroy et al., 2018). Early marriage and childbirth are high with about 60% of women age 20–24 marrying and close to half of all Bangladeshi women giving birth by 18 years of age and before reaching physical maturity (National Institute of Population Research and Training [NIPORT] et al., 2016).

There has been a dearth of research investigating adolescent eating behaviours and their determinants in Bangladesh. The few studies conducted have shown that limited dietary diversity, suboptimal energy and micronutrient intake, and undernutrition are higher in the lowest wealth quintiles, with household wealth associated with the probability of better nutritional status (Akhter & Sondhya, 2013; Alam, Roy, Ahmed, & Ahmed, 2010; Leroy et al., 2018; National Institute of Population Research and Training [NIPORT] et al., 2016). Data from rural areas indicate that education and empowerment of female household heads are insufficient to improve nutritional status of adolescents, suggesting that resources may be too constrained (Leroy et al., 2018). To our knowledge, potential contributions of more distal social and cultural factors contributing to adolescent food intake in Bangladesh have not been explored.

Gender has proven to be a key criterion when assessing decisions related to intrahousehold food allocation (Haddad, 1999). Research conducted in Nepal found that women were systematically not allowed to consume more expensive, prestigious foods (Gittelsohn, Thapa, & Landman, 1997). A study carried out in India showed that discrepancies in the value attributed to males and females caused discrimination in the allocation of foods for girls (Das Gupta, 1987). In Ethiopia, study results demonstrated that preferential treatment of adolescent boys led to gender‐based differentials in food insecurity experienced by girls (Hadley, Lindstrom, Tessema, & Belachew, 2008).

Using gender as an overarching framework, qualitative research was conducted to examine individual, social, environmental, and structural factors influencing eating behaviours of female adolescents living in low‐income families in urban and rural settings in Bangladesh.

## 2 RESEARCH METHODS

### 2.1 Study setting and population

**Figure 1:**
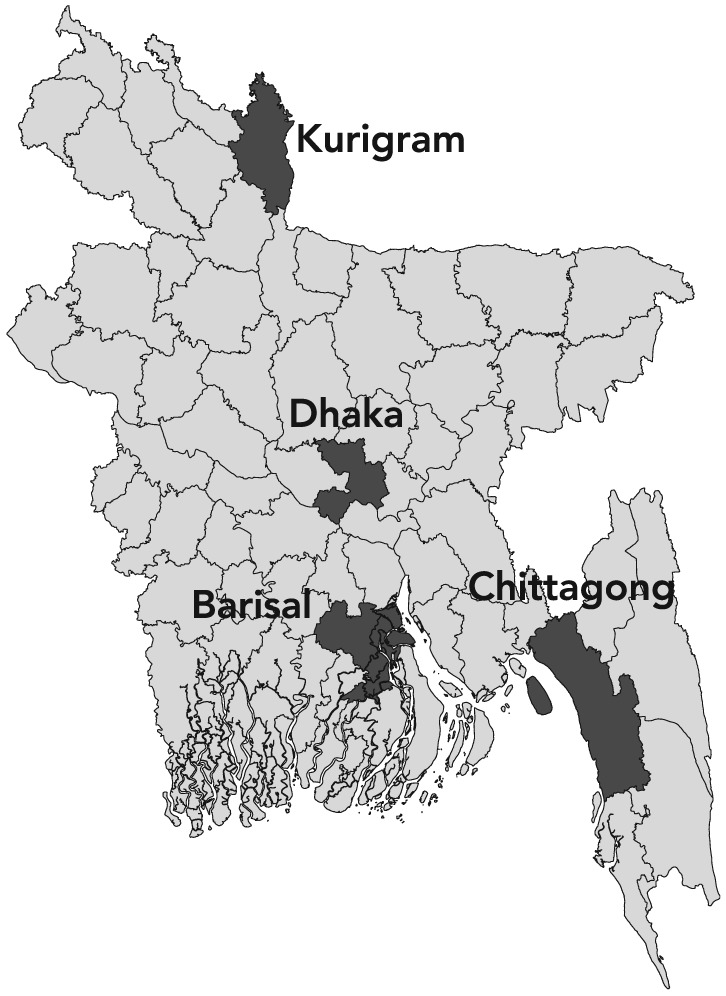
Map of Bangladesh indicating the districts where the study was conducted

Data collection was carried out between April and June 2017 in urban slums in the cities of Dhaka and Chittagong and rural areas in the districts of Barisal and Kurigram (see Figure 1). Selection of research sites was designed to include low‐income urban and rural settings that differed geographically, topographically, and culturally. Dhaka and Chittagong, the most populated cities in Bangladesh, are infiltrated with slums inhabited by immigrants in search of cash employment. Barisal is a southern fishing and agricultural district located on flood plains, and Kurigram, a northern farming district, is one of the poorest and most food insecure regions of Bangladesh.

### 2.2 Study design, sampling, and methods

Two study areas in each research site were included, with slums in Dhaka and Chittagong selected randomly. In Kurigram and Barisal, one village close and one far from the main district town were selected purposively. Adolescent respondents ranged from 15 to 19 years of age and were divided into three categories typical of this age group, including students, girls who had discontinued school, and married adolescents. We only sampled unmarried girls residing with their parents. The research used a mix of qualitative methods administered across the four research sites as described below.

*Free‐listing exercises* were employed to assess the cultural domain of meals. School‐going girls were identified on the road while going to school or in their homes, whereas non‐school‐going and married adolescents were approached near or in their households. The initial target was to conduct 30 exercises.

*Key informant interviews* were conducted with professionals working directly or indirectly on issues related to adolescence at the national level or in research communities to examine determinants of eating behaviours. Key informants were selected purposively based on their expertise and knowledge of adolescent health, nutrition, and social behaviour, with the goal to continue interviewing until reaching data saturation.

*In‐depth interviews* were administered with school‐going, non‐school‐going, and married adolescents. The aim was to explore individual motivations for food consumption, patterns of food intake, environmental and social factors guiding eating behaviours, and the social context of eating. We also examined perceptions of health and work responsibilities. School‐going girls were identified in or nearby schools, whereas the non‐school‐going and married respondents were approached in households with the target to interview eight females representing each of the different adolescent categories. Prior to in‐depth interviews, day‐long direct observations of subsamples of in‐depth interview respondents were conducted. Observations started in the morning and followed respondents' activities throughout the day with the objective to assess meal and snack food consumption, as well as household cooking patterns and food distribution. We also observed work and school schedules and social interactions. Observation data were referred to when conducting in‐depth interviews with the same adolescent respondents.

*Group discussions* were administered with fathers and mothers of adolescent females ages 15–19 years, as well as adolescent males 15–19 years of age. During discussions, we explored determinants of food acquisition and consumption, perceptions of intrahousehold food distribution, and household decision‐making and responsibilities. Participants were selected with assistance from community key informants and opinion leaders, with the aim to include six to 10 participants in each group.

### 2.3 Data collection procedures

Data were collected by five research assistants, two supervisors, and an anthropologist responsible for field activities. Prior to data collection, a 5‐day training on data collection methods and ethical procedures was held in Dhaka. Study instruments were tested in a slum and modified based on the field experience.

Teams of reseach assistants travelled to the study sites sequentially, starting in Dhaka and subsequently going to Barisal, Kurigram, and Chittagong. In each site, initial data collection involved freelisting exercises and key informant interviews. Key informant interviews were open‐ended, designed to last approximately 1‐hr, and typically conducted in the informant's work place. A guide was used, with ensuing questioning incorporating emerging study findings. Subsequently, adolescents were selected for observations and in‐depth interviews. Research assistants employed a form to record observational data. A semi‐structured guide was followed to conduct in‐depth interviews, with interviews limited to 1.5 hrs. When needed, researchers returned to households for additional information. Group discussions lasting no longer than 2 hrs were held at the end of data collection in each site. A guide based on study themes and preliminary results of other research methods was used.

All interviews were carried out in Bengali, the Bangladesh national language. Key informant and in‐depth interviews and group discussions were audio recorded. Efforts were made to maintain privacy when administering all data procedures.

Research investigators were in regular communication during data collection to discuss study results and modifications of instruments. A debriefing meeting of investigators was held after completion of data collection.

### 2.4 Data analysis

Freelisting exercises were analysed on Anthropac 4.983 software (Borgatti, 1996). Salience was derived using a salience index (Smith's *S*) defined as *S* = ((*L* − *Rj* + 1)/*L*)/*N*, where *L* is the length of each list, *Rj* is the rank of item *J* in the list, and *N* is the number of lists in the sample (Borgatti, 1999). Items with a higher salience score were considered to have greater significance (Bernard, 1988; Weller, 1984). The audio recorded interviews and observational data were translated into English from Bengali and transcribed. Transcripts were reviewed by senior researchers during the course of the study. After data collection, a coding system was developed for key informant, in‐depth interviews, observations, and group discussions based on reviews of transcripts. Coding categories were derived from initial research themes, as well as emerging concepts. Coding of interview transcripts was done on ATLAS.ti, a text‐organizing software (ATLAS.ti Scientific Software Development, 2013), whereas observational and group discussion data were coded in matrixes. Content analysis was used to identify trends of concepts in and across individual codes. The combination of data, environmental and methodological triangulation, facilitated data analysis across the different research sites and methods and across and between key informants and adolescent respondents.

### 2.5 Research ethics

The study protocol was reviewed and ethical approval granted by the Institutional Review Board Bangladesh Medical Research Council, Dhaka, Bangladesh. Verbal informed consent and assent from guardians of respondents under 18 years of age were obtained from all freelisting and in‐depth interview respondents, key informants, and focus group participants prior to data collection.

## 3 RESULTS

### 3.1 Background information

We administered 33 freelisting exercises across the research sites. Seven key informants composed of community‐based health workers, school teachers, and opinion leaders were interviewed in study communities. National level informants included two NGO representatives working on adolescent and maternal health and nutrition, a researcher with gender expertise, and a social marketer. Eight adolescents from each respondent category participated in in‐depth interviews, with five or six of each observed. Observations lasted from about 7 a.m. to 5 p.m. Due to security and logistic reasons, research assistants were unable to observe evening meals, which often occurred late at night. In each site, we administered discussions with separate groups of mothers and fathers of adolescent girls and adolescent boys. Fathers were involved in income‐generating activities, whereas mothers were working women and housewives. Adolescent boys were school‐going, working, and nonworking. Table 1 presents the number of respondents according to each data collection method.

**Table 1:** Data collection methods and numbers of informants according to the research site

Method	Type of respondent	Research site				No. (each type)	Total no.
		Dhaka	Chittagong	Barisal	Kurigram		
Free‐listing exercise	Girls age 15–19 years	8	8	8	9	N/A	33
Key informant interviews	School teacher	—	—	1	1	2	11
	Community leader	—	—	1	—	1	
	Community health worker	1	1	—	2	4	
	National level expert	4	—	—	—	4	
In‐depth interviews	School‐going	2	2	2	2	8	24
	Non‐school‐going	2	2	2	2	8	
	Married	2	2	2	2	8	
Observations	School‐going	1	1	2	1	5	16
	Non‐school‐going	1	2	1	1	5	
	Married	2	1	1	2	6	
Focus group discussions	Mothers	1	1	1	1	4	12
	Fathers	1	1	1	1	4	
	Adolescent boys	1	1	1	1	4	

The average age of adolescents in respondent categories was between 16 and 18 years (Table 2). All respondents were Muslim. School‐going and non‐school‐going adolescents had more years of education than those in the married group, with one married respondent never attending school. Mean months of marriage was 15.5; two married respondents had children, and one was pregnant.

**Table 2:** Socio‐economic background of the adolescent in‐depth interview respondents

Variables	School going (*n* = 8)	Non‐school‐going (*n* = 8)	Married (*n* = 8)
Average age (in years)	15.8	16.1	17.5
No. of household members			
No education	—	—	1
1–5	1	1	3
6–10	7	7	3
10+	‐	‐	1
No. of household members			
Average	6	8	5
Range	(4–10)	(5–12)	(2–9)
Family type			
Nuclear	5	3	4
Extended	3	5	4
Family wage earners			
One person	4	3	5
More than one person	4	5	3

Half of households where adolescent respondents lived had more than one earning member, particularly those in cities. Most urban households had women engaged in wage labour, whereas females residing in rural sites were not earning money. One school‐going and one married respondent generated small wages through home‐based manufacturing activities. Common occupations of male household heads were small business, day labour, driver, and service employee, such as garment worker or security guard. No urban‐based families possessed land in their home districts. All families in rural areas owned homestead land and about half had small agricultural plots where they produced rice for consumption.

### 3.2 Food items and eating patterns

#### 3.2.1 Freelisting

Exercises identified 87 foods eaten during meals with rice, fish, green leaves, lentil, egg, vegetables, and potato the most salient. Typical of a cultural domain, procedures elicited a core set of more significant and a wide range of less important meal items, with the top 15 ranked foods listed in Table 3. Vegetables and types of fish were meal foods most frequently listed.

**Table 3:** Most salient meal foods identified by adolescent females during freelisting exercises

Item no.	Bengali name	English name	Frequency	Average rank	Smith's saliency
1	Bhat	Rice	33	1.0	1.000
2	Maach	Fish	31	3.7	.786
3	Sobuj shakshobjee	Green leafy vegetables	30	7.3	.580
4	Daal	Lentil	23	5.2	.517
5	Dim	Egg	24	7.1	.485
6	Shobjee	Vegetables	20	5.0	.446
7	Alu	Potato	24	9.4	.392
8	Mangsho	Meat	15	5.1	.355
9	Potol	Pointed gourd	21	11.7	.259
10	Vendi/dherosh	Okra	16	10.1	.246
11	Murgi mangsho	Chicken	19	13.2	.246
12	Panggash maach	Cat fish	17	11.8	.234
13	Kumra	Pumpkin	16	12.8	.218
14	Lau	Bottle gourd	16	14.1	.207
15	Torkari	Curry	7	2.1	.195

#### 3.2.2 Eating patterns of the female adolescents

At the time of the study, most adolescents reported eating three meals daily, although some from Kurigram claimed their families ate twice. Prior to breakfast, some families offer a “light” meal consisting of biscuits, crispy rice (*chaal bhaja*) 1Uncooked rice is fried in a hot pan, which makes the rice crispy. or puffed rice (*muri*) for members rising early. Adolescents described family meals as dominated by rice with curry 2Curry is usually a dry or gravy dish which includes one or more vegetable items cooked with spices and oil. Sometimes driedfish is mixed with the vegetable, but this is mostly for taste. Curry can also be combined with a pulse. dishes, with most families eating either fish or eggs daily. Chicken is eaten once weekly or bi‐monthly, often Friday, with beef consumed only on special occasions.

We observed that all respondents consumed breakfast, with school‐going girls eating earlier in the morning (Table 4). Breakfast most often consisted of leftover rice with curry, with a few adolescents also eating meat or fish. Some respondents substituted rice for *roti* (traditional flatbread made of whole meal flour) eaten with curry or lentils. One girl only ate rice with chilli. Food portions differed from those of male members who ate full plates of rice or several pieces of *roti* with curry, whereas the girls ate small amounts of rice or one or two pieces of *roti* with or without curry. Several school‐going girls claimed to skip breakfast often, stating they did not like leftovers in the morning.

**Table 4:** Food intake during observations of the different categories of adolescent females

Meal and consumption		Type of informant		
		School‐going (*n* = 5)	Non‐school‐going (*n* = 5)	Married (*n* = 6)
Breakfast	No. who ate	5	5	6
	Timing	6:00 a.m.–11:00 a.m.	6:30 a.m.–9:45 a.m.	8:00 am–11:15 a.m.
	Food content	• Rice, chilli: 1 • Rice, mashed potato: 2 • *Roti*, lentil, curry: 1 • Biscuit, tea: 1	• Rice, vegetable curry: 2 • Rice, fish, meat: 1 • *Roti*, tea/curry: 2	• Rice, vegetable curry: 3 • Rice, vegetable curry, fish or chicken: 2 • *Roti*, tea: 1
Lunch	No. who ate	5	5	6
	Timing	2:30 p.m.–5:00 p.m.	1:00 pm–3:00 p.m.	1:30 pm–3:30 p.m.
	Food content	• Rice, fish: 2 • Rice, chicken: 1 • Rice, mashed potato, lentil: 1 • Rice, vegetable curry: 1	• Rice, vegetable curry, lentil: 2 • Rice, fish: 2 • Rice, egg, lentil:1	• Rice, vegetable curry: 2 • Rice, chicken: 2 • Rice, three kinds of meat, fish: 1 • Rice, fish, curry, lentil: 1
Snacks	No. who ate	1	2	3
	Timing	10:30 am	11:00 am–12:00 am	10:30 am–11:30 am
	Food content	• Rice with chilli	• Two biscuits: 1 • Soda:1	• Mango, sweetmeat, sugarcane: 1 • Mashed mango: 1 • Rice with vegetable curry: 1

Lunch was consumed between 1:00 and 5:00 pm, with those eating a late breakfast consuming lunch later in the day. School‐going adolescents ate lunch late in the afternoon after returning home from school; this was sometimes the first food eaten since breakfast. During observations, most respondents ate fish or animal source foods for lunch, whereas the others consumed vegetable curry and lentils with rice. One married respondent who attended a special festivity consumed different meats and fish. Although foods consumed varied little with other family members, we observed that portions of fish or meat were less than that given to males. An exception was the breastfeeding respondent whose food portions were observed to be comparable with those of her husband. We observed one married respondent refuse to eat chicken her husband intentionally placed on her plate.

Respondents reported that dinner is eaten between 8 pm and midnight. Many respondents claimed to have little appetite at dinner time, eating small amounts when the meal is comprised of leftovers or less desirable foods such as green leafy vegetables, missing dinner particularly when lunch is consumed late in the afternoon, or falling asleep before dinner is served.

Adolescents indicated that snacks generally entail leftover meals, packaged or street foods such as *chanachur* (a spicy dry mix) and biscuits, or homemade rice‐based items. Snacks were described as “light” foods eaten in small amounts between meals to appease hunger or when meal foods are unavailable, the meal is delayed, or for breakfast. Few respondents were observed eating snacks between meals. Those snacks consumed were limited in portions or quality (Table 4). However, during interviews, over half of the respondents claimed to eat snacks regularly between meals, particularly leftovers or puffed rice, when they are hungry. A couple of married respondents reported occasionally storing packaged biscuits at home to address hunger or as a dinner substitute. Most school girls received pocket money occasionally from parents to purchase snack foods.

Most respondents reported frequently experiencing hunger between meals, with married respondents emphasizing hunger between lunch and dinner. Adolescents across all groups reported periodically eating meals consisting of rice with chilli.

### 3.3 Gender constraints on food consumption

This section highlights ways in which gender norms define male and female roles, which in turn influence food acquisition, decisions surrounding intrahousehold food allocation, consumption patterns, and discrepancies related to actual food intake, placing adolescent females at risk for poor health and undernutrition.

### 3.4 Economic hardship and food insecurity

Parents participating in focus groups stated that food purchasing is driven by food availability and especially cost. Most adolescent respondents and group discussion participants reported that staple foods such as rice, potato, onion, and lentil are purchased in bulk weekly or less frequently, whereas several families in Kurigram and newly married nuclear families relying on low‐paying and irregular daily wages bought staples daily. Families in rural areas producing rice for family consumption reported periodic shortages. We were told that fresh vegetables are generally obtained on a daily basis, particularly green leaves, which can be purchased for little money, or in rural settings, gathered. Due to the high price, fruit is only bought on special occasions. Eggs are purchased multiple times weekly, with rural households also raising chicken for eggs. Fish is procured once or more weekly, but meat is rarely bought due to the cost. According to key informants, low‐income families buy chicken organs, legs, and skin discarded by hotels and sold in markets near slum communities as a meat substitute. Focus group participants indicated that male household heads are responsible for obtaining staples, animal food sources, and vegetables, whereas females may buy less costly curry items. Women in Kurigram were prohibited from going to the market. Fathers reported purchasing fish and vegetables in night markets on the way home from work when foods are cheaper, with some admitting to buying rotting food items.

Adolescents living in rural households and most newly established nuclear married respondents reported food shortages occurring during deficits in rice production or the rainy season when day labour generates less money, causing reductions in meal consumption and alterations in content, with meals sometimes consisting of rice or packaged snacks. Extended families in urban areas described periodic modifications in meal quality precipitated by financial constraints linked to reduced daily wages and salary payment schedules with more vegetable‐based meals and eggs eaten during economic hardship. Correspondingly, special purchases are more frequent when day laborers garner extra payment or at the start of the month after salaried workers receive wages. Some parents of unmarried girls reported occasionally honouring requests for costly items such as meat and fruit, particularly from boys and younger children, whereas in homes of married respondents, in‐law's or husband's food choices were prioritized. Fish or meat is sometimes obtained by borrowing money or through credit with shopkeepers.

Only respondents living in more solvent households reported storing packaged snacks. Girls provided occasional pocket money during school obtained foods costing on average 10 taka (approximately 0.8 USD), claiming the quantity was not enough to stave off hunger. Some filled up with water, as reported by this girl (IDI_16),

(When there is nothing) I walk around and drink water. That's it. I manage to fill my stomach by drinking water. Because I feel hungry, but I cannot tell my mother this. She does not have money.

Across sites, cooking took place once or twice daily, with females universally in charge. Cooking three meals was too time consuming and expensive, with rural respondents underlining the cost of firewood and food ingredients. Slum dwellers must stand in line to use kitchen facilities shared with 10–12 families. In these settings, adult females involved in income‐generating activities were unable to wait a long time to cook. These factors forced many slum dwelling families to cook once daily.

Families cooking once a day often prepared food around noon with the same meal consumed for lunch, dinner, and breakfast the following day when dishes are reheated and eaten with watery rice. If there are no leftovers, breakfast consists of foods requiring little or no preparation such as *roti*, biscuits, and *chira*. When cooking occurs twice, one meal is prepared early in the morning and consumed for breakfast and lunch by family members at home. A second meal is cooked in the evening or night. Several respondents from Kurigram and married adolescents in nuclear households reported that dinner is cooked late at night when male wage earners return home with food, with married respondents often forced to skip lunch and to wait as late as midnight to prepare the dinner meal and eat. In Kurigram, this was generally the only meal cooked during the day.

#### 3.4.1 Household food distribution

Key informants, adolescent respondents, and group discussion participants indicated that women and adolescent girls receive less food than males, citing multiple explanations. Females usually eat after other members have been served or have eaten. We observed that often little of the curry item is left, forcing the person serving and other females to eat less. When the accompanying curry dish is limited, rice portions are also reduced. This father (FGD_2) said,

It often happens that little food remains after distributing to others, and they (the mother and her daughters) eat whatever is left in the pot. Sometimes no food remains.

A married respondent (IDI_23) said,

My mother‐in‐law and my husband eat first. I eat later. Sometimes there is (food) and sometimes not. When something remains, I eat and when nothing is left, I do not eat. When there is nothing, I take some light foods. I eat chaal bhaja (crispy rice).

Married respondents maintained that because they live in their in‐law's home, do not generate an income, and spend the day performing chores perceived menial, it is normal to receive less food. Key informants reported that particularly if the dowry has not been paid, new brides are the least prioritized in regard to food intake. Some married respondents were reluctant to request more or special food or even eat with others, often choosing to miss the dinner meal when family members eat. Group participants indicated that adolescent females perceived to eat excessively are subject to ridicule, especially when married and living with in‐laws, thus inhibiting them from eating much or requesting preferred food. One married respondent (IDI_20) said,

In the in‐laws you are not able to eat the same way as in your own house (paternal home). Sometimes you remain hungry. You do not eat out of shyness. Brides feel shy; they think what the father‐in‐law and mother‐in‐law might think … They might say the girl eats huge amounts, things like this. What do I do? I remain hungry, eating less.

During observations, we found married respondents to be demure and obedient towards their in‐laws and husband. Several married respondents reported being victim to verbal and emotional abuse inflicted by their mothers‐in‐law and husbands, and in one instance, the mother‐in‐law periodically restricted food as punishment.

Due to their economic roles, bigger physiques, and perceptions that males are more active, men and adolescent males were reported to be given both larger quantities and better‐quality foods, with boys also favoured because they are expected to provide money and care for their parents in old age. This non‐school‐going girl (IDI_6) said,

They are men. They have more rights. They work hard and earn. We eat from their earnings … So if there is a food shortage, my mother, my sister‐in‐law and I split the food among us.

A married adolescent (IDI_24) stated,

My husband and my father‐in‐law get more food. They work hard. They are men. They have to be given more. My mother‐in‐law and I work hard, but we have to give more to men. This is obvious. My mother told me this before my marriage.

Group discussion participants suggested that from childhood, boys are socialized to believe they need more food, whereas girls are expected to leave the household shortly after completing primary education and thus receive less preferential treatment. One father (FGD_5) said,

Daughters need to be married off when they grow up. Sons are considered essential for the future. Sons will provide food and shelter when they grow up but daughters leave after marriage. For this reason, love goes a little more to sons than daughters.

As they get older, boys were reported to demand food by showing anger or refusing to eat if they considered meals insufficient. One boy (FGD_6) stated, “I will feed my parents by earning money after I grow up, which is why I make demands (related to food) on them.” Group discussion participants contended that daughters are more cognizant of family economic limitations and have lower expectations regarding their personal needs. This father (FGD_11) said,

Daughters are affectionate and willing to make sacrifices like their mothers. They understand the sufferings and limitations of the family.

Key informants agreed that cultural norms enforce gender‐based eating differences, explaining that females are encouraged to give more and special foods to male members and expected to eat less during food shortages and financial hardship. One key informant said,

From a very young age girls are taught to make sacrifices, and if you do not sacrifice, you are not a girl. Girls learn about this from the adolescent period. In food insecure households, if they are short in meals, first the adult woman gives up her meal and then the teenage girl.

Many adolescent respondents expressed satisfaction for being able to sacrifice food so that fathers and brothers have bigger portions, whereas serving more or better food was a way of showing affection towards husbands. Some national key informants underscored that discrimination towards girls is changing, emphasizing that birth order, number of female children, and household size influences the way females are valued.

#### 3.4.2 Daily schedules, household responsibilities, and mobility

School girls followed a structured schedule attending school and tutoring from early in the morning. They returned home late in the afternoon when some reported that the better lunch portions like fish might no longer be available. School‐going girls performed household tasks after school and on weekends. Unmarried, non‐school‐going girls started and ended the day doing chores, with breaks particularly in the late afternoon, when they watched television or chatted with friends living nearby. Married adolescents worked in the household from morning to evening or nighttime when dinner is served. Those living in extended households had more responsibilities due to numbers of family members. Much of their day involved responding to ongoing requests, particularly by the mother‐in‐law, father‐in‐law, and husband, and ensuring their comfort. Household chores across adolescent respondents included food preparation, serving meal foods, washing dishes, fetching water, sweeping, washing clothes, and providing childcare for younger siblings or, in the case of two married respondents, their own children.

Working household members left early in the morning, with some men returning home around dinner time. Aside from going to school, most unmarried girls were permitted to leave the neighbourhood only when visiting relatives or on holidays and accompanied by a family member. Restrictions were intended to reduce exposure to lewd remarks, protect girls from sexual or physical abuse, and maintain their reputations, with parents indicating that girls circulating neighbourhoods freely are viewed negatively. One school girl (IDI_15) said,

My father does not allow me to go anywhere … He does not even allow me to go to school picnics … he worries a lot. I am a girl and he worries about my safety …

Another non‐school‐going girl (IDI_4) said,

As a child, I used to move freely. I do not do this anymore. People will say bad things and therefore I do not walk around the neighbourhood.

Unmarried girls lamented that recent restrictions in their mobility prevented them from playing. Those no longer in school described other lifestyle changes, claiming they have become sedentary, cautious when interacting with people, particularly men, and engaged in household work; all of which made them feel prematurely grown up.

Married respondents had to abide to harsher restrictions, specifying that their husbands prohibit them from venturing beyond the immediate household surroundings. They described pressure to maintain family dignity, which prevented them from interacting with nonfamily members, with most claiming not to have friends nearby. This respondent (IDI_18) said,

The situation has changed. Before I visited here and there. After marrying, if I do that, people will talk behind my back. When I get hurt, whom can I complain to? I do get hurt.

Observations confirmed that most married adolescents did not leave the household and had limited contact with other people. Many desired the comfort provided by natal family members and social interactions with friends. Married respondents did, however, occasionally visit their natal family.

#### 3.4.3 Gender expectations, nutrition and health

Most adolescents expressed concerns about their health. Unmarried respondents commonly complained of fatigue, weight fluctuations, dizziness, and social tension, claiming that health problems interfere with studying and going to school, as well as performing household chores. These respondents often attributed inadequate food intake to small meal portions, long gaps between meals, and poor appetite, particularly when given frequently served meal foods and during menstruation. Tensions related to looking attractive and subscribing to the modest behaviour expected of adolescent girls, as well as concerns about their future schooling, falling in love and maintaining family dignity, and marriage, were also perceived to reduce appetites. School‐going girls worried about having to terminate school due to costs, exposure to sexual harassment while travelling to school, or an arranged marriage, with all strongly opposed to marrying young. Although unmarried girls no longer in school preferred postponing marriage, many predicted marrying soon and voiced concerns of being victim to violence inflicted by in‐laws, causing tension. Key informants suggested that adolescent girls are keenly aware of parents' efforts to preserve their social reputations, which can be tarnished if adolescents engage in relationships with males or are victims of sexual abuse. A primary concern relates to increased dowry costs as the girl ages. One community key informant said,

Social pressure and security are bigger problems than providing food (to girls at home). For families and parents, having a girl at home who has left school is a big social issue. When a girl sits idle at home, people expect she will be married off as soon as possible. Families cannot ignore such pressure.

The few girls claiming good health attributed their well‐being to proper food intake.

Married respondents were more vehement about health problems, with six of eight asserting their health had deteriorated since marriage, which they associated with reduced appetite and food intake and weight loss, with all admitting feeling hungry regularly. Women who had conceived or had a child expressed heightened concerns due to added physical demands. Psychological stresses associated with married life involved tenuous household economics, being the subject of scrutiny, gossip and criticism, and expectations regarding caring for family members and performing household chores. One respondent (IDI_19) said,

Now I think about my husband. If he gets ill there will not be any income. I have grown thinner. My health used to be good. But now my body has become thin due to stress.

Many expressed remorse for marrying, namely, that they lost independence, the possibility of earning money, and personal dreams, with some expressing feelings of despondency and dejection. When talking about nonmarried peers, one respondent (IDI_17) said,

I do not have the same situation, I mean, I do not have that age. If I had, I could do a lot of things. I could study more. I could attain a good position in life. I am not like other girls. They can go outside, meet with many people, but I cannot. I am a daughter‐in ‐law in this house.

## 4 DISCUSSION

Study findings illuminated how gender norms interact with economic, social, and structural factors to make female adolescents vulnerable to poor dietary intake. Intrahousehold food allocation involving serving males first and bigger food portions limits the amount of meal foods reserved for adolescent and adult females, which were consistently small. Both male and female study participants maintained that adult males require more food because of their larger physique and critical financial roles. Household chores performed by females are considered menial, contributing to perceptions that females have smaller food needs. Younger males are prioritized due to their social capital as future wage earners and caregivers. In contrast, girls are expected to marry and join another family. Social scientists have examined the inferior value attributed to females living in low‐income households who require a dowry, typically do not generate an income, and are considered an economic burden until they leave the household (Chowdhury, 2004; Parsons et al., 2015). In preparation for marriage, Bangladeshi girls are socialized to adhere to cultural principles associated with a good woman which involves being modest, obedient, and respectful, characteristics attractive to potential suitors (Chowdhury, 2004; Schoen, 2015). Female socialization involves making sacrifices, which in this study entailed consuming smaller food portions so that male family members ascribed with higher value can eat more. Respondents gained satisfaction by offering food to males, acknowledging family economic constraints and their less consequential status.

In the context of low‐income households, the interaction of food insecurity and prevailing gender norms works synergistically to limit dietary intake. Biologically, adolescent males have increased energy requirements compared with females the same age, and in food secure settings, boys would be expected to eat larger food portions (Spear, 2002). However, in these food insecure environments, inequities in food distribution based on gender (not biological sex differences) result in females given inadequate food to meet their nutritional needs. Although favouring males in or expected to enter the paid work sector may seem from the families' perspective to be a logical coping strategy, our results suggest that gender discrimination in the allocation of limited household food resources makes adolescent females more vulnerable to hunger and malnourishment. Other research carried out in South Asia has suggested that economically active family members and those expected to contribute to the household welfare in the future receive preferential treatment in relation to food resources (Gittelsohn, 1991; Senauer, 1990). Low female status has been shown to impact on household decision‐making involving resource allocation, thus limiting women's ability to influence important familial decisions related to health and nutrition and perpetuating the intergenerational cycle of undernutrition (Haddad, 1999). Many studies have demonstrated that maternal education is positively associated with nutritional status in children and adolescents (Madjdian, Azupogo, Osendarp, Bras, & Brouwer, 2018; Makoka & Masibo, 2015). Although increasing education should improve female status in Bangladesh, changes will depend on the quality of schooling and subsequent opportunities for income‐generating activities. As was the case in our study, Bangladeshi adolescent females living in poor families have low secondary school attendance, are still marrying early, and generate little if any income once married (National Institute of Population Research and Training [NIPORT] et al., 2016), negating the possibility of modifying their position in society. It is not clear how more education, which delays marriage age, impacts the dowry price and the social prospects of adolescent girls. Traditionally, age increases investments required by the bride's family (Amin & Huq, 2008). Whereas family prestige, social pressure, and government programmes encourage female schooling, the dowry may be higher for better educated females, who may also be considered older prospective brides (Field & Ambrus, 2008; Parsons et al., 2015; Schuler, Bates, Islam, & Islam, 2006; Streatfield, Kamal, Ahsan, & Nahar, 2015). Social tenets opposing premarital sex and maintaining family dignity and concerns about female safety also foster early marriage (Chowdhury, 2004; Rashid, 2006; Schuler et al., 2006; Streatfield et al., 2015).

Limited food intake was most apparent among newly married females who are considered strangers when they enter their in‐law's household. Married respondents, who claimed to be subject to scrutiny and criticism, developed strategies such as skipping meals, eating alone, consuming small amounts, and sacrificing food portions for others, all aimed at meeting expectations regarding their family role and avoiding attention. Low incomes and limited assets in newly formed households further restrict food consumption of married adolescents. The combination of their young age, perceived inferior sex, and economic dependence make new brides highly vulnerable to discrimination and suppression, which in turn affects food intake, as elucidated by married adolescents in our sample. Another study conducted in Bangladesh reported limited decision‐making power and economic assets affect access to food resources by newly married adolescent women (Nguyen et al., 2017). Social status changes with the arrival of a first baby, marking acceptance as a family member (Rashid, 2006). In our study, the only married woman regularly provided more and special food items was a mother who reported significant changes in her treatment after conception and childbirth.

Work and school schedules fostered long breaks between meals, with the study highlighting minimal consumption of snacks to ease hunger. School‐going girls who are away from home for extended stretches have less access to leftovers available between meals or were unable to afford snacks that filled them up while at school. Male household heads either provide money or assume the role of food purchasing, thus influencing the timing of meal preparation and consumption. More financially constrained families forced to purchase foods daily postponed consumption of the dinner meal until late at night after earning members returned home. All three types of adolescent respondents reported skipping meals, particularly dinner, because they ate a late lunch (school‐going girls) or fell asleep before dinner is served. Married respondents in nuclear households were forced to skip lunch if their husbands were away during the day and no food was available in the household. Other studies have reported skipping meals, eating smaller portions, and consuming only rice during meals as coping strategies followed by adolescent girls during food insecurity (Helen Keller International [HKI] & James P. Grant School of Public Health [JPGSPH], 2016), which appeared to be frequent occurrences in our sample, underlining the commonality of these practices.

The combination of poor food intake and strenuous work schedules may explain why many respondents complained of ill health, with most asserting that their limited food consumption does not meet their physical requirements. A common complaint was lack of appetite with some describing meal foods as undesirable. Sociocultural norms and economic factors force adolescent females to assume adult roles, leading to a variety of psychological stresses including concerns about growing work responsibilities, their impending marriage, and for married respondents, household finances at a prematurely young age. Married respondents claimed a deterioration in their health since marriage, mentioning a multitude of socioemotional and physical stresses, including unhappiness, weight loss, social isolation, and abuse in the form of food deprivation. Limitations on mobility and interpersonal interactions deprived adolescents of entertainment and social outlets, leading to social isolation. These restrictions are linked to *purdah*, rules designed to limit interactions between males and females and maintain gendered behaviour (Chowdhury, 2004; Schoen, 2015). Numerous studies show increased risk to common mental health conditions (Gebreyesus, Endris, Hanlon, & Lindtjørn, 2018; Hadley & Patil, 2006), weight loss and dietary modifications (Rose, 1999; van Liere, Ategbo, Den Hartog, & Hautvast, 1995), and poor health in food insecure households (Casey et al., 2005; Cook et al., 2004; Cook et al., 2013; Siefert, Heflin, Corcoran, & Williams, 2004). A study in the United States found that food insecurity among adolescents was associated with chronic depression and contemplation of suicide (Alaimo, Olson, & Frongillo, 2002). In Bangladesh, gender discrimination and its manifestations likely amplify susceptibility to psychological conditions among adolescent girls living in food insufficient households.

In conclusion, findings illuminate sociocultural and economic factors that constrain food intake in adolescent females at a time when nutritional requirements are elevated. On a continuum, newly married adolescents, who have more demanding workloads and are expected to bear children, were the most extreme example, with married respondents consistently eating irregular, small food portions. In order to improve the nutritional status of adolescent girls, both gender norms and food insecurity influencing dietary intake must be recognized and addressed. In Bangladesh, the prevalence of adolescent marriage and pregnancy is among the highest in the world, signifying the strength of social and economic factors that guide early marriage and roles relegated to women (Loaiza & Liang, 2013; UNICEF, 2017). Policies and programmes must continue to be implemented to increase marriage age and reduce adolescent pregnancy. In addition, the government and its partners need to develop programmes that ensure that school‐going children and adolescents eat adequate nutritious foods during the school day. Such efforts should ensure that nutritious foods are available in and around school settings and include educational outreach to parents and teachers. Research examining psychological health and food intake among adolescents merits exploration. Over the long‐term, increasing job opportunities, prolonging school education, and strengthening the economic viability of young women should influence the value ascribed to girls and women and thus alter cultural expectations regarding age at marriage and initiation of childbearing and normative female roles in households and society. Increased female empowerment should impact on household decision‐making, including decisions related to food intake of adolescent girls.

## CONFLICT OF INTEREST

The authors declare that they have no conflicts of interest.

## AUTHOR CONTRIBUTIONS

LSB, RK^1^, YS, SS, RK^3^, and AT designed the study. RK^1^ supervised field operations, and RK^1^, MS, and NS conducted data collection. LSB, RK^1^, MS, and NS carried out data analysis and interpretation. This paper was written by LSB and RK^1^ with substantial input from all other authors. All authors have reviewed and approved the submitted manuscript.
